# Models of visual word recognition

**DOI:** 10.1016/j.tics.2013.08.003

**Published:** 2013-10

**Authors:** Dennis Norris

**Affiliations:** Medical Research Council Cognition and Brain Sciences Unit, 15 Chaucer Road, Cambridge CB2 7EF, UK

**Keywords:** reading, computational modelling, lexical decision, word recognition

## Abstract

Reading is a complex process that draws on a remarkable number of diverse perceptual and cognitive processes. In this review, I provide an overview of computational models of reading, focussing on models of visual word recognition–how we recognise individual words. Early computational models had ‘toy’ lexicons, could simulate only a narrow range of phenomena, and frequently had fundamental limitations, such as being able to handle only four-letter words. The most recent models can use realistic lexicons, can simulate data from a range of tasks, and can process words of different lengths. These models are the driving force behind much of the empirical work on reading. I discuss how the data have guided model development and, importantly, I also provide guidelines to help interpret and evaluate the contribution the models make to our understanding of how we read.

## Highlights

• I review models of visual word recognition and data used to evaluate them.
• I focus on recent IA and mathematical/Bayesian models.
• I explain how models process and represent letter order.
• I suggest how competing models should be evaluated.

## From boxes and arrows to computational models of reading

Reading is an impressive human achievement that requires coordinated mastery of a constellation of perceptual and cognitive processes ranging from low-level visual perception to recognition of word forms, phonological processing, eye-movement control, and all of the higher-level linguistic processes required to recover the meaning of the written words. Understanding each of these processes is hard but understanding how they operate as a whole presents an even greater challenge. Early models of reading were predominantly of the ‘box-and-arrow’ type. However, even the most influential of these models – Morton's logogen model [1] – had very little to say about exactly what went on in the boxes or what information flowed along the arrows. The situation changed dramatically with the development of computational models of reading in the early 1980s. These models made clear statements about what was supposed to be going on in the boxes and we could now work out exactly what the models predicted. These first models were simple connectionist networks (see Glossary). Since then, models have increased in their ability to produce ever-more-accurate simulations of an increasingly wide range of challenging data. New models continue to emerge, with several of the more recent models departing from the connectionist tradition. This review concentrates primarily on more recent models that try to explain the core process that uniquely characterises reading: recognising words as visual objects. These visual objects can then make contact with the full range of representations in the reader's mental lexicon.

## Why ‘computational’ models?

Models of reading are almost invariably computational models. This is true of theories of word identification 2, 3, 4, 5, 6, 7, 8, 9, 10, 11, reading aloud 12, 13, 14, 15, morphology [16], and eye movements in reading text 17, 18, 19 and of models of spoken word recognition 20, 21. How has the field come to be so reliant on computational models? After all, in many cases the underlying principles of the models are simple. However, even with a deep understanding of the principles and mathematical foundations underlying the models, it is almost impossible for theorists to be sure how their models will behave. The reason is straightforward: the behaviour of the models is not determined simply by the high-level theoretical principles themselves, but emerges as an interaction between those principles and the contents of the lexicon. How any one word will be processed depends critically on the nature of the other words in the lexicon. Given that some of the models now use lexicons containing many tens of thousands of words, the only way to be sure exactly what the theories predict is to implement them as computational models. However, although there is universal agreement that computational models are to be preferred over older verbal or box-and-arrow models (logogen), there is a continuing debate about the most useful style of model.

## Modelling style

**Figure 1:**
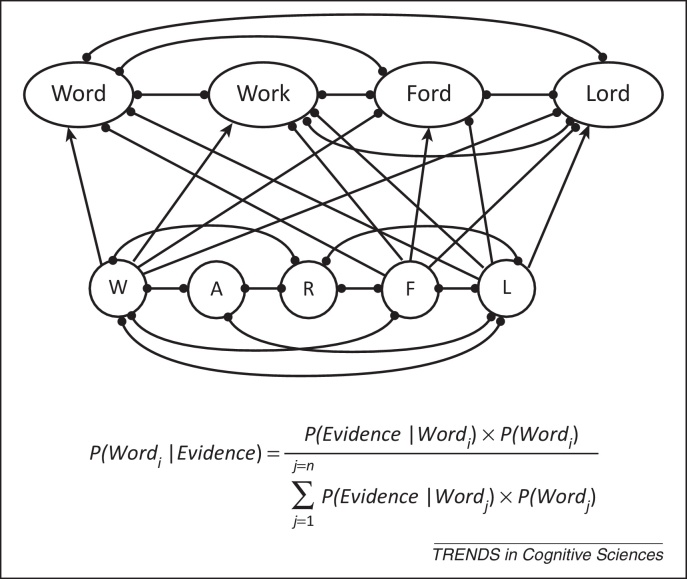
Different styles of model. The top panel illustrates a simplified interactive activation model. Lines with arrows denote excitatory connections from letters to words. The lines terminated with circles denote inhibitory connections. Similar words (lexical neighbours) compete via these inhibitory connections. In a Bayesian formulation, words also compete; if the probability or likelihood of one word increases, the probability of other words must decrease. The network and mathematical approaches are much more closely related than they might first appear. Note that the Bayesian formulation must necessarily take account of the prior probability of each word; that is, its frequency.

The earliest and most influential style of computational model is the interactive activation (IA) model 11, 22 (Figure 1) – one of the first connectionist or ‘neural-network’ cognitive models. In almost all IA models, letter features, letters, and words are represented as nodes in a network (a ‘localist’ representation). IA networks generally have no capacity to learn. Although IA models remain popular (the Spatial Coding Model [2] and the dual-route cascaded (DRC) model [12] are two recent examples in this tradition), many connectionist models incorporate learning mechanisms and use ‘distributed representations’. This is most common in models of reading aloud 15, 23, 24, 25. In models using distributed representations, words are usually not represented by a single node, but as a pattern of activations over a set of nodes.

Connectionist models are often favoured because they appear ‘brain like’ [26] or ‘neurally inspired’ [27]. An alternative view is that we know so little about how words might be represented in the brain, or how the relevant neural computations are performed, that we should formulate our models at a more abstract level that makes no claims about implementation and concentrate instead on understanding the nature of those computations [28]. Many of these models are therefore expressed primarily in terms of computational procedures or mathematical formulae. Table 1 lists the most influential computational models and indicates which style of modelling they use and the primary phenomena they have been developed to explain. Note that although the primary focus here is on models of visual word recognition, the table lists a broader range of models, including connectionist models of reading aloud and models of eye-movement control in reading.

**Table 1:** Major computational models of reading organised in terms of their primary focus^a^, ^b^

Model	Style	Task	Phenomena	Large lexicon
**Models of visual word recognition**				
IA 11, 22	IA	PI	Word-superiority effect	
Multiple read-out [3]	IA	PI, LD	Word-superiority effect	
SCM [2]	IA	LD, MP	Letter order	
BR 4, 5, 6	Math/comp	LD, MP	Word frequency, letter order, RT distribution	√
LTRS [8]	Math/comp	MP, PI	Letter order	
Overlap [66]	Math/comp	PI	Letter order	
Diffusion model [30]	Math/comp	LD	RT distribution, word frequency	
SERIOL [7]	Math/comp	LD, MP	Letter order	
**Models of reading aloud**				
CDP++ [13]	Localist/symbolic	RA	Reading aloud	√
DRC [12]	IA	RA, LD	Reading aloud	
Triangle 24, 25	Distributed connectionist	RA	Reading aloud	
Sequence encoder [15]	Distributed connectionist	RA	Reading aloud	√
Junction model [50]	Distributed connectionist	RA	Reading aloud	√
**Models of eye-movement control in reading**				
E-Z reader 17, 18	Symbolic	R	Eye movements	
SWIFT [19]	Symbolic	R	Eye movements	
**Model of morphology**				
Amorphous discriminative learning [16]	Symbolic network	Self-paced reading, LD	Morphology	√

a The table also indicates the modelling style or framework, the main task that the model simulates, the main phenomena that the model simulates (not exhaustive), and whether the model uses a realistically sized lexicon. Note that the review concentrates on ‘Models of visual word recognition’.

b Abbreviations: Math/comp, mathematical or computational; LD, lexical decision; PI, perceptual identification; RA, reading aloud; MP, masked priming; R, natural reading.

When comparing different styles of model, appearances can be deceptive. For example, Ratcliff's drift–diffusion model (DDM) 29, 30 is usually expressed mathematically, but can trivially be recast as a simple connectionist network if the network is crafted to compute exactly the right function. That would change how the model looks, but would not alter the underlying theory or explanation. Similarly, Figure 1 contrasts an IA model with Bayes formula, which is the basis of the Bayesian Reader (BR). However, IA models can also be formulated to compute Bayes theorem. Next, I give a brief description of the three most recent models of visual word recognition 2, 6, 8 that also illustrates contrasting modelling styles. One is a connectionist model and the other two are mathematical/computational.

**Figure 2:**
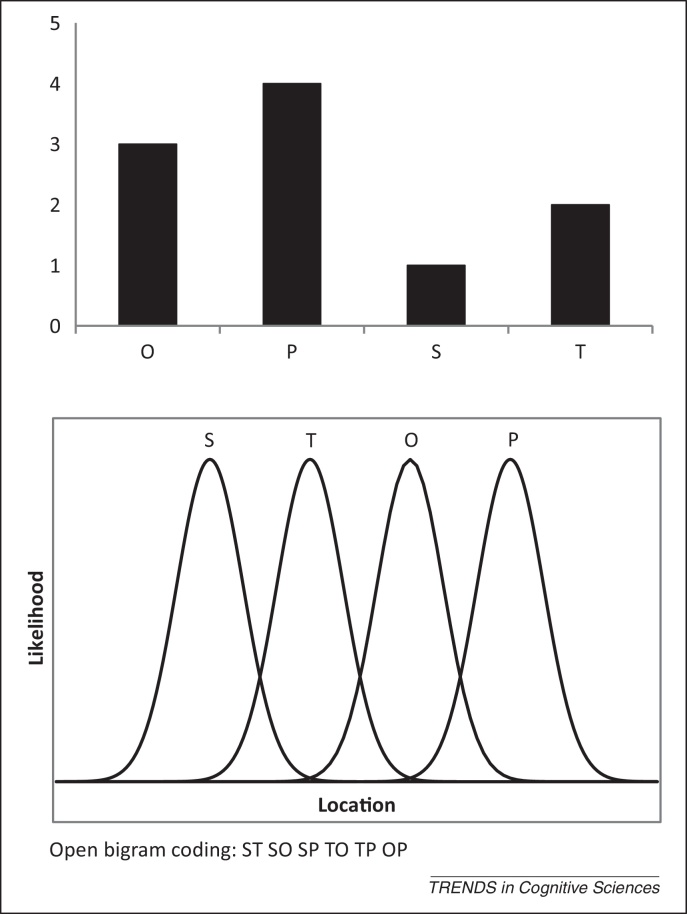
Three different representations of letter order. The Spatial Coding Model (top) represents letter order as a gradient of activation over letter nodes that increases with letter position. The noisy channel and overlap models (middle) both assume that there is some uncertainty in the location of letters. That is, there is some probability that T might have come before S. Open-bigram models (bottom) code letter order as a set of bigrams.

The Spatial Coding Model (SCM) [2] is based on the IA framework. The original IA model could simulate words of a fixed length only. The SCM has been further developed to enable it to process words of varying lengths and to simulate masked priming. The distinctive feature of the SCM model is the way that it represents the order of letters in the input in terms of an activation gradient over letter positions (Figure 2). The model incorporates a matching rule that is relatively insensitive to exactly where words begin in the input (TOP will be activated in STOP) and also tolerates minor changes in the relative position of letters (JUGDE will activate JUDGE; see Box 2).

The Letters in Time and Retinotopic Space (LTRS) [8] model was developed primarily to account for data on perceptual identification and masked priming. It assumes that information about letter identity and letter order accumulates stochastically over time. Importantly, although there is variability in the time at which a letter is identified, letters and their associated order information are always identified correctly. Given the prime JUGDE in a masked priming task, there is some probability that, at the end of the prime, the only evidence that has accumulated might be JU*GE, where * corresponds to one or more unknown letters. This would be consistent with the word JUDGE and produce priming. However, given the prime JUNPE, if either N or P are identified this will be inconsistent with the target and not produce priming (Box 2).

The LTRS model makes no specific assumptions about the precise form of representations – any representation will do, providing it has the correct set of properties. As Adelman notes [8], several representations would satisfy the requirements – for example, a representation involving letters and open bigrams – but the specific choice of representation makes no difference to the model predictions. Indeed, an important contribution of LTRS is to show how a wide range of priming data can be explained while making few assumptions about the exact form of representations.

As with the LTRS model, the BR [6] is formulated at an abstract level that makes no assumptions about implementation and as few assumptions as possible about representations. The aim of the BR is to see how much can be explained simply by assuming that readers make near-optimal decisions based on the accumulation of noisy evidence. The model is optimal in the sense that, for a given level of accuracy, it will identify words based on the fewest number of samples possible; that is, as fast as possible [31]. Bayes theorem provides the optimal procedure for combining uncertain evidence with knowledge of prior probability.

In the model, letters are represented as vectors describing coordinates in a multidimensional space. The dimensions could be considered to correspond to letter features, although they also encode positional information. At each time step, the model accumulates a noisy sample from the input that is created by adding noise to the input vector. As more samples are accumulated, the model's estimate of the true value of the input becomes more precise and hence the identity and position of the letters is known with greater certainty. For each word in the lexicon, the model computes the likelihood of observing the input, given that the word would have generated that input *P*(*evidence*|*word*). The model also knows the frequency of each word *P*(*word*). From this, it can use Bayes theorem (Figure 1) to compute the probability of each word given the input *P*(*word*|*evidence*). A word can be identified when this probability exceeds some predetermined threshold. In a connectionist model, all words will have some degree of activation. In the BR, activation becomes something much more specific: probability. To focus on the core principles, the model incorporates many simplifying assumptions about the nature of the visual information available. For example, all letters in a word are assumed to be equally perceptible.

In the BR the focus is on optimal decision making. This means that the model naturally accounts for differences between tasks such as lexical decision, perceptual identification, and masked priming. Different tasks require different decisions; therefore, the optimal decision process must necessarily be different, too. Additionally, a Bayesian model must necessarily take account of prior probability, which gives a natural explanation for the word frequency effect. As already noted, the way any one word is processed depends on its relation to other words in the lexicon. The way that words influence each other is generally viewed as a process of lexical competition.

## Lexical competition

To recognise a word, the reader must accumulate enough evidence to distinguish that word from perceptually similar words: their lexical neighbours. Perceptually similar words must compete with each other for recognition. All current models incorporate some form of lexical competition, although the way that competitive process operates can appear to be different in models that produce very similar behaviour (Box 1 and Figure 1). They also incorporate different assumptions about the form of the perceptual and orthographic representations of words and the way they are processed. Words that are considered to be close neighbours in one model might not be in another 32, 33. This is most apparent in the way different models make contrasting assumptions about the way letter order is represented (Box 2).

Box 1 **Styles of modelling: IA models versus Bayesian theories**

IA models (see Figure 1 in main text) have several appealing features. One is that they are relatively easy to understand. The basic principle is one of competition between word nodes. Words receive activation in proportion to how well they match the input and nodes compete with each other by means of inhibitory connections. The best-matching word will win the competition but be slowed down by competition from similar words. The most advanced IA model is the SCM [2]. The SCM differs from earlier IA models in that it can deal with words of different lengths. This allows it to simulate a far wider range of phenomena than earlier models. One concern with IA models is that the networks generally require many parameters whose exact values have no principled motivation. For example, how much inhibition should there be between words or how should the models implement the effect of word frequency? In a Bayesian model 6, 9, 12, such questions do not arise; the precise treatment of lexical competition and word frequency follows automatically from the theoretical claim that readers approximate ideal Bayesian decision makers. Figure 1, in main text, shows a simple IA model and Bayes theorem. Although a connectionist network and an equation look like very different things, they achieve similar ends. Each word node in the IA model sums its perceptual input from letter or feature nodes. Because each word node is connected to every other node, all nodes receive the same amount of inhibition, where that inhibition is proportional to the sum of all other nodes. According to Bayes theorem, the probability of each word is a function of the evidence for that word (called the likelihood) divided by the evidence for all other words. There is a clear parallel between the two formalisms. Consequently, a properly configured network could compute Bayes theorem, but it could also compute a range of different functions. Would we gain anything by implementing a Bayesian model as a connectionist network? A network implementation would simply compute exactly the same function and produce exactly the same simulations, but it would make it harder to appreciate the importance of the theoretical claim that readers were approximating ideal Bayesian decision makers. As Anderson [28] noted, ‘If two theorists propose two sets of mechanisms in two architectures that compute the same function, then they are proposing the same theory’.

Box 2 **The representation of letter order**

As witnessed by the ease with which we can read the famous ‘Cmabrigde Uinervtisy’ email (http://www.mrc-cbu.cam.ac.uk/people/dennis.norris/personal/cambridgeemail), readers are remarkably tolerant of changes in the order of letters in a word 70, 71. For example, in the masked priming task, a prime constructed by transposing two letters of the target word produces as much priming as an identity prime (*jugde*–JUDGE versus *judge*–JUDGE) and much more than a prime where the same two letters are changed (*junpe*–JUDGE) 72, 73, 74, 75. This excludes the simplest possible theory of letter coding in which letters have position-specific codes. Under that scheme, a ‘d’ in position 4 is a completely different entity from a ‘d’ in position 3, so *jugde* should produce no more priming than *junpe*. Figure 2, in main text, illustrates three alternative letter-coding schemes. Open Bigram coding appeals to a form of local-context coding using pairs of letters 32, 76, 77, 78. JUDGE and *jugde* are deemed to be very similar because they share nine of ten open bigrams, whereas JUDGE and *junpe* share only three. In models using noisy coding of position or order 6, 66, 68, 79, 80, letter order is simply represented as a sequence of letters, as would be found in a dictionary. JUDGE and *jugde* are similar because uncertainty over the exact position of the letters means that the noisy perceptual input generated by *jugde* could have been produced by *judge*. In the SCM, order is represented as a gradient of activation over letter nodes [2]. Studies have shown that priming can also be produced when letters from the target are deleted or other letters are inserted 75, 78, 81, 82, 83. There are now three computational models that can generate very accurate simulations of most of the experimental data 2, 6, 8, but none of these relies on open bigrams. All of these models appeal to some form of noisy sampling or noisy coding. One major challenge for open bigram models is to explain how it is that *fo* primes OF [84]; given that the prime and target do not have any open bigrams in common, there should be no priming. By contrast, this result is exactly what would be expected from all of the other models.

Almost all studies of neighbourhood effects use Coltheart's N [34] as a measure of neighbourhood density. This metric considers only words of equal length to be neighbours. However, words of different lengths such as ‘hat’ in ‘that’ also act as competitors 35, 36, 37, 38. A better measure of density that accounts for more unique variance in lexical decision times is provided by the orthographic Levenshtein distance (OLD20) [39]. The Levenshtein edit distance is given by the number of edits (insertions, deletions, and substitutions) required to transform one word into another. The OLD20 is based on the average edit distance of the 20 nearest neighbours.

## Laboratory tasks

Although the goal of models of word recognition is to understand normal reading, the only overt behaviour that readers generally produce is to move their eyes. Eye-movement data are hugely informative, but it is rarely practical to collect large amounts of data using carefully controlled stimuli. Many researchers therefore turn to more tractable laboratory tasks such as lexical decision, word naming, and masked priming. This leads to two distinct modelling enterprises. Whereas models of eye-movement control during reading tend to make simplifying assumptions about how individual words are identified 17, 18, 19, models of word recognition rarely consider how they might be integrated with models of reading. The use of laboratory tasks poses an additional modelling challenge. Although it is tempting to think of tasks like lexical decision as being direct measures of the time taken to identify a word, each of the tasks engages some additional task-specific processing. For the models to fit the data, they must simulate task performance as well as word identification itself.

Fortunately, the results tend to be similar in research in which the same phenomena have been studied using eye movements and lexical decision 40, 41, 42. However, there is one area where different tasks do produce different results. As noted above, all current models incorporate some form of lexical competition; words with many neighbours should therefore be recognised more slowly than words with few neighbours because they suffer from more competition 38, 43. However, in lexical decision this pattern is reversed [39]. In IA models 2, 3, 12, this finding is an embarrassment because the networks just have to predict that recognition will be slowed by competition. To overcome this problem, the models have to be modified by adding a decision process that is sensitive to the overall activation in the lexicon 3, 12. More neighbours produce more overall activation which leads to faster responses. They can then account for the opposite pattern of data from the one they naturally predict.

This situation highlights an important contrast between theories that begin by postulating a particular mechanism 2, 11, 22 and those that focus instead on higher-level computational principles 4, 5, 6. For IA models, the problem is that their explanation of word recognition lies in the details of the mechanism. If the behaviour changes, the mechanism must somehow be changed, too.

In the BR 4, 5, 6, the optimal decision must necessarily differ between different tasks. In a perceptual identification task, participants need to select one word from among all the words in the lexicon. In lexical decision, the participant's task is not to select a single word, but to press a button when they are confident that the input is a word rather than a nonword. The optimal way to respond is to pool the evidence over all words that are similar to the input [4]. Words in dense neighbourhoods will therefore be responded to faster in lexical decision. The BR has to predict that neighbourhood effects will vary depending on the task. They should be facilitatory in lexical decision but inhibitory in tasks requiring identification of a unique word. This follows directly from the idea that readers approximate ideal Bayesian decision makers. By contrast, IA models naturally produce inhibition. To fit the data they can be modified to produce facilitation, but they do not explain why different tasks should produce different results.

## The rise of the megastudy

Until relatively recently, the standard recipe for a study of reading would be to carefully select small subsets of words that varied on one or two measures of interest and then to compare them using either a lexical decision task or a speeded naming task. However, we now have access to several large-scale databases, or megastudies, containing lexical decision data for between ten and 40,000 words. The largest of these, the English Lexicon Project (ELP) [44], contains 4 million word-recognition trails collected from over 1200 participants. Data for the ELP was collected in the USA, but there are now similar databases for British English [45], Dutch [46], and French [47]. The ELP also contains data on naming as well as lexical decision. Eye movement data is available from the Dundee corpus [48], which was derived from ten English and ten French participants each reading about 50,000 words. Many hypotheses can therefore be tested by performing virtual experiments on the databases.

Keuleers *et al.* [45] performed several such experiments where they compared item reaction times (RTs) from previous experiments on word frequency, regularity, feedforward consistency, age of acquisition, polysemy, and neighbourhood density with corresponding item RTs in the British Lexicon Project (BLP). In some cases, the BLP data did not show the same effects as in the original studies. Perhaps the theoretically most significant ‘failure to replicate’ in these virtual experiments was that the BLP did not consistently reveal a facilitatory effect of neighbourhood density (Coltheart's N). However, the BLP does show the expected correlation with measures of neighbourhood density, albeit a slightly smaller correlation than that seen in the ELP [6]. All of the megastudies show a similar pattern. Yap and Balota [49] presented an analysis of both lexical decision and naming latencies of 6115 monomorphemic multisyllabic words from the ELP. They examined the influence of a range of measures including word frequency, letter and syllable length, phonological and orthographic neighbourhood density, and spelling-to-sound consistency. These factors accounted for about 61% of the total variance in both naming and lexical decision.

The rise of the megastudy has raised the bar in terms of what we expect from our computational models. Why stop at just being able to simulate the effect of, say, spelling-to-sound regularity or neighbourhood density using a small set of carefully controlled stimuli? Now we can ask how well the models can simulate item-level RTs for all of the words in the databases. Modellers have started to rise to this challenge. Yap and Balota [49] analysed simulated data from Kello's [50] junction model and Perry *et al.*’s [13] Connectionist Dual Process (CDP++) model (see Table 1 for more information on these models) in the same way that they had analysed the human data. With some exceptions, they found that both models were sensitive to the same factors as were human readers. The CDP++ model [13] of reading aloud has been used to simulate reaction times for over 32,000 words, 17,841 of which were in the ELP. The BR [6] simulates lexical decision times for over 26,000 words from the ELP and most of the words in the British, Dutch, and French lexicon projects. Other models simulate smaller but still substantial portions of the megastudy items 2, 51, 52. Although the megastudies are an invaluable resource, they have limitations. Whether for lexical decision or for reading aloud, the correlations between the megastudies, or earlier smaller-scale studies, never exceed 0.7 13, 45. This is not greatly different from the split-half correlations in the BLP [45]. Lexical decision and naming data are fundamentally noisy [53]. Even the same subjects will respond differently on different occasions 54, 55. The studies use different equipment and different nonwords and even vary regarding whether words are presented in upper case (ELP) or lower case (the British, Dutch, and French lexicon projects). Even more importantly, they use different participants with different linguistic experience. The most obvious consequence of this variability between megastudies is that there is an upper limit on how much variance we can expect models to account for. Current models can achieve correlations of about 0.6 with human RTs. Given that the maximum correlation between megastudies is only about 0.7, it might appear that there is only limited room for improvement. However, this does not mean that the models are so good that they cannot be developed further. For example, currently none of the models has the ability to simultaneously model orthographic, phonological, and semantic effects.

The megastudies confirm that the single most powerful determinant of lexical decision or naming speed is the logarithm of the word's frequency of occurrence in the language (although there is debate about the exact form of this function 56, 57). So how do the models explain the word-frequency effect? Most models simply build the effect in without offering any explanation of why things should be that way. For example, connectionist learning models almost always present words during training in proportion to the logarithm of their frequency, not their actual frequency 13, 50. However, a Bayesian model must take account of prior probability (Figure 1); that is, its frequency. When this is combined with the assumption that perception involves the accumulation of noisy evidence, this automatically produces the observed logarithmic relation between frequency and RT [4]. That is, the Bayesian model delivers the log frequency function for free and this explains why we should observe a logarithmic function rather than any other.

## Beyond mean RT: simulating variability

The usual target for models of word recognition is mean RT. However, even more information can be extracted from the data by examining the distribution of RTs and how they change as a function of stimulus type 58, 59, 60, 30, 61, 62 or participant group 63, 64. IA models therefore always respond to the same word in exactly the same way and in exactly the same amount of time. This means that they are unable to simulate RT distributions (although see [65]). By contrast, evidence-accumulation models 4, 5, 6, 8, 30, 66, 67, 68 start from the assumption that perception is a fundamentally noisy process and that the task of the perceptual system is to make the best use of that noisy information. The most successful of these models is Ratcliff's DDM 29, 69, which is usually applied to two-choice RTs and can therefore be used to model RT distributions in lexical decision. In the DDM, evidence is accumulated as a sequence of noisy samples until the total evidence reaches a ‘yes’ or ‘no’ decision boundary. The DDM gives a very accurate fit to a range of lexical decision data [30] and provides some interesting insights. For example, it was shown that word frequency influenced the rate at which evidence was accumulated. Norris [5] showed that this pattern follows directly from the BR's account of word frequency. Note that some of these data can be simulated in an IA model by adding a leaky accumulator decision process to the output [65].

## Concluding remarks

Modelling word recognition began with small-scale simulations using perhaps a thousand words, all of the same length [22]. The target for simulation was perceptual identification scores from a few small datasets. Models can now perform large-scale simulations of data from tens of thousands of words. The scope of the models has been expanded to cover tasks like lexical decision, masked priming, reading aloud, and eye-movement control. Models now simulate a far wider range of empirical findings than their predecessors and some can simulate RT distributions as well as means. Although, comparing models is rarely straightforward (Box 3), much of the empirical work in the area is now targeted at testing differential predictions of the models. Despite their successes, current models all have limitations (Box 4). In particular, individual models tend to focus on a single domain of behaviour, such as reading aloud, eye movements, or lexical decisions. There is a need for more integrated theories of word recognition.

**Glossary**

**Bayes theorem:** a mathematical procedure for updating probabilities or beliefs in the light of new evidence. In the case of word recognition, the probability of a word given the input, or evidence, is as follows:
  $$P(word|evidence)=P(word)\times p(evidence|word)/\sum_{i=0}^{i=n}\left[P(word_{i})\times p(evidence|word_{i})\right]$$
**Connectionism:** models expressed as artificial neural networks; this includes, for example, the IA model .These models are intended to capture general properties of neurons, or neuronal populations.
**Interactive activation (IA) model:** the first, and still most influential, form of connectionist model of word recognition. Words are represented as nodes in a network that are connected by inhibitory links (see Figure 1 in main text).
**Lexical competition:** in both IA models and Bayesian models, neighbouring words compete with each other for recognition. In IA models, this is due to the inhibitory connections between word nodes.
**Lexical decision:** the most common laboratory task for studying word recognition. Participants are required to decide whether a string of letters is a word or not (a nonword).
**Masked priming:** a variant on the lexical decision task in which the target is preceded by a briefly presented prime, which can be a word or a nonword. Participants are rarely aware of the prime. The prime is usually presented in lower case and the target in upper case to minimise physical overlap. Masked priming is most commonly used to address questions about the representation of orthography.
**Neighbourhood density:** a measure of how similar a word is to other words. A common measure is Coltheart's N [34]: how many other words can be formed by changing a single letter in a word? According to this definition, only words of the same length can be neighbours. A more flexible measure is given by a Levenshtein distance metric. This measures similarity in terms of the number of ‘edits’ – insertions, deletion, and substitutions – so WORD and WORDS will now be considered to be neighbours. The OLD20 is the average distance of the 20 closest neighbours.
**Open bigrams:** a proposal that the order of letters in a word is coded in terms of a set of ordered letter pairs, which may be non-contiguous. WORD might be coded as WO, WR WD, OR, OD, or RD
**Reaction time (RT) distribution:** RTs in tasks like lexical decision are generally positively skewed. Variables like word frequency rarely shift only the mean of the distribution, but usually the form of the distribution, too. Accounting for these changes is a challenge for computational models.
**Word-frequency effect:** by far the strongest influence on how readily a word can be identified is its frequency of occurrence in the language; words that occur very often in the language are recognised more quickly than low-frequency words. The speed and ease with which words can be recognised is an approximately logarithmic function of word frequency.

Box 3 **Evaluating models**

Given the wide range of computational models available, how should we set about evaluating them? What makes one model better than another? The usual selling point of a model is to emphasise how well it fits the data. A model that cannot fit the data is clearly of little value. However, neither is a model that can fit any pattern of data that might possibly be observed [85]. A partial solution to this problem is to use formal methods for comparing models with different numbers of free parameters 86, 87, 88 that penalize models with greater flexibility. However, sometimes flexibility does not come from the settings of free parameters but from *ad hoc* modifications to the structure of the model designed to accommodate new pieces of awkward data. Of course, in itself, extending and developing models is no bad thing, but models should be ‘nested’ 89, 90 such that any new version should still be able to simulate the data covered by the old model. Given that old models evolve and new models need to fit the data to be published, models tend to converge.

Perhaps the most important question to ask of any model is whether it provides a good explanation of the data. A computer program that happened to simulate the data but whose operation was opaque would make little contribution to our understanding of word recognition [91]. The model should be a computational implementation of a theory and the explanation is a property of the underlying theory rather than the model [92]. We need to look beyond the particulars of the model and ask how the principles and assumptions of the theory explain how words are recognised. Ideally, we would also like a theory to shed some light on why our perceptual processes operate in the way they do. Indeed, addressing the why question is one of the main goals of the Bayesian approach 93, 94. Box [95] famously stated that ‘all models are wrong, but some are useful’. A model that is wrong but useful may be better than a model that is ‘right’ (fits the data) but of little use in helping to explain the phenomena of interest. We should value models for their theoretical insights and not just for their ability to fit the data.

Box 4 **Outstanding questions**

• Current models each deal only with subcomponents of the reading process. One of the greatest challenges is to produce an integrated model of reading. For example, we have no process models of how morphological or semantic representations interact with orthographic processing.
• Many of the challenges facing models of reading are shared with all models of visual perception. For example, we know little about how readers achieve translational invariance; that is, the ability to recognise words presented in different locations or to recognize morphemes embedded in longer words (e.g., like in dislike). How are ‘a’, ‘A’, and ‘a’ all treated as instances of the letter ‘a’?
• How is word recognition influenced by differences between languages and writing systems?
• Can we make models more relevant to the understanding of reading disorders? Current computational models of word recognition concentrate on fluent reading in the stable adult system and have little to say about how reading develops and how that development might be impaired.
